# CD133 Expression in Placenta Chorioangioma Presenting as a Giant Asymptomatic Mass

**DOI:** 10.3390/medicina57020162

**Published:** 2021-02-11

**Authors:** Gianluca Di Massa, Guglielmo Stabile, Federico Romano, Andrea Balduit, Alessandro Mangogna, Beatrice Belmonte, Pina Canu, Emma Bertucci, Giuseppe Ricci, Tiziana Salviato

**Affiliations:** 1 Department of Diagnostic, Clinic and Public Health Medicine, University of Modena and Reggio Emilia, 41125 Modena, Italy; gianluca.dimassa@unipr.it (G.D.M.); pinacanu@tiscali.it (P.C.); salviato.tiziana@aou.mo.it (T.S.); 2 Institute for Maternal and Child Health, IRCCS Burlo Garofolo, Via dell’Istria, 65/1, 34137 Trieste, Italy; guglielmo.stabile@burlo.trieste.it (G.S.); federico.romano@burlo.trieste.it (F.R.); giuseppe.ricci@burlo.trieste.it (G.R.); 3 Department of Life Sciences, University of Trieste, 34127 Trieste, Italy; abalduit@units.it; 4 Tumor Immunology Unit, Department of Health Promotion, Mother and Child Care, Internal Medicine and Medical Specialties, University of Palermo, 90134 Palermo, Italy; beatrice.belmonte@unipa.it; 5 Prenatal Medicine Unit, Obstetrics and Gynecology Unit, Department of Medical and Surgical Sciences for Mother, Child and Adult, University of Modena and Reggio Emilia, 41125 Modena, Italy; emma.bertucci@unimore.it; 6 Department of Medical, Surgical and Health Science, University of Trieste, 34129 Trieste, Italy

**Keywords:** placental chorioangioma, giant asymptomatic mass, CD133 expression

## Abstract

*Background*: Placental chorioangioma is the most common benign non-trophoblastic neoplasm of the placenta. Its clinical relevance lies in the size of the tumor since larger masses cause pregnancy complications, including an unfavorable neonatal outcome. *Case presentation:* We report the case of a 34-year-old second gravida and nullipara at the 35th week of gestation, admitted to the gynecological department for antibiotic-resistant fever. The cardiotocography performed during hospitalization showed an abnormal fetal pattern. A 2250 g newborn was delivered by cesarean section. No complications were observed during childbirth and postpartum was insignificant. On gross inspection a white fleshy intraparenchymal mass blooming on the maternal surface was noted; routinely stained sections revealed features consistent with chorioangioma with vascular channels lined by inconspicuous endothelial cells immunoreactive for CD31 and CD133. Focal expression of CD133 was also observed in placental villi. *Discussion:* CD133 expression indicated the presence of stem cells in chorioangioma, suggesting their possible role in the development of mesenchymal lesions including chorioangioma.

## 1. Introduction

Placental chorioangioma is a fetal-stromal vascular lesion, being part of a wider subgroup of lesions, called villous capillary lesions, which includes the following variants characterized by excessive angiogenesis: chorioangiosis (presence of more than 10 capillaries per terminal villus in at least 10 villi within several placental regions), chorioangiomatosis (represented by small anastomosing capillaries mostly in the tip of placental stem and immature intermediate villi), chorioangioma (similar to chorioangiomatosis for stem and immature intermediate villi involvement but different for the formation of a nodular expansile lesion) and multiple chorioangioma syndrome, an infrequent variant with several concomitant chorioangiomas occupying up to 80% of the total volume of placental parenchyma [1,2,3,4]. Chorioangioma with trophoblast proliferation is an additional condition to be accounted for differential diagnosis with the histological forms mentioned above. This is a rare proliferation of trophoblastic tissue, usually exhibiting nuclear atypia and pleomorphism, which may be suggestive of a malignant condition in close association with chorioangiomas. In this case, however, trophoblasts may be recognized in virtue of their “multilayering” aspect through Ki-67 detection, highlighting a differential pattern from traditional chorioangioma [5].

The first vascular tumors were described by Clarke in 1798 [6,7]. In terms of incidence, chorioangioma occurs in about 1% of all pregnancies [6,8,9,10], being considered as the most common benign tumors of the placenta. Three histological patterns have been described by Marchetti [7,11]: angiomatous, cellular and degenerate. The angiomatous pattern is the most frequent and consists of a proliferation of capillaries and small vessels surrounded by chorial villi. In the cellular pattern, the stroma is looser and there is an abundant proliferation of endothelial cells. The degenerate pattern shows calcification, necrosis or hyalinization areas [12]. Small chorioangiomas (<5 cm) are usually asymptomatic and are either not diagnosed or incidentally found at a histological examination; large or giant chorioangioma, variously defined as measuring more than 4–5 cm, are often diagnosed prenatally and their prevalence ranges from 1 in 9000 to 1 in 50,000 pregnancies [7,13].

The main purpose of our report was to demonstrate the expression of CD133 in placental chorioangiomas which have never been described before. We believe that the widespread positivity of CD133 in chorioangioma may be suggestive of stem cell involvement in its formation.

## 2. Case Report

A 34-year-old woman, *secundigravida* nullipara, was admitted to our Obstetrics and Gynecology Unit of the Policlinico Hospital of Modena at the 35th week of gestation because of an antibiotic-resistant fever. A rapid swab test for SARS coronavirus 2 was negative. She was hemodynamically stable. Pertinent laboratory examinations resulted within the normal limits, and other infections were ruled out. Ultrasound examinations of placental plate and placental annexes were unremarkable and fetal biometry was within the ranges. However, a pathological cardiotocography tracing was observed and subsequent hospitalization was required to perform an urgent cesarean section. A vital 2250 g newborn (Apgar score 8 at 1 min and 9 at 5 min) was delivered.

**Figure 1:**
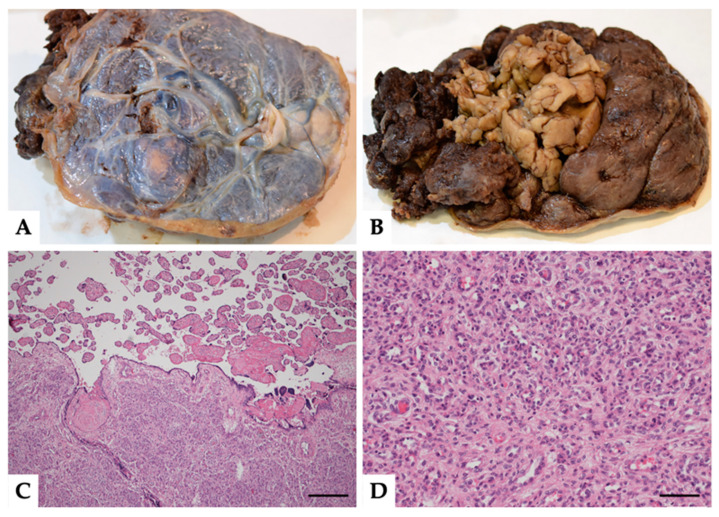
(**A**,**B**) Gross specimen of the placenta. Fetal surface (**A**) and maternal surface (**B**) with the gross appearance of the fleshy mass. (**C**,**D**) Histopathological findings of the placental chorioangioma. Panoramic view of abrupt transition between normal parenchyma and chorioangioma ((**C**) magnification 100×, scale bar 200 μm, hematoxylin and eosin, H&E). Particular of the chorioangioma at higher magnification: a network of small and medium-size vessels, lined by endothelium cells with inconspicuous nuclei and without atypia chorioangioma ((**D**) magnification 200×, scale bar 100 μm, H&E).

Placenta, membranes and umbilical cord were sent for histological examination. On gross inspection, the fixed placenta specimen measured 17 × 14 cm and weighed 406 g, showing a 6 cm white fleshy intraparenchymal mass blooming on the maternal surface (Figure 1A,B). Histologically, the mass was composed of a dense and convoluted network of small and medium-size vessels, lined by endothelium cells with inconspicuous nuclei and without atypia (Figure 1C,D).

**Figure 2:**
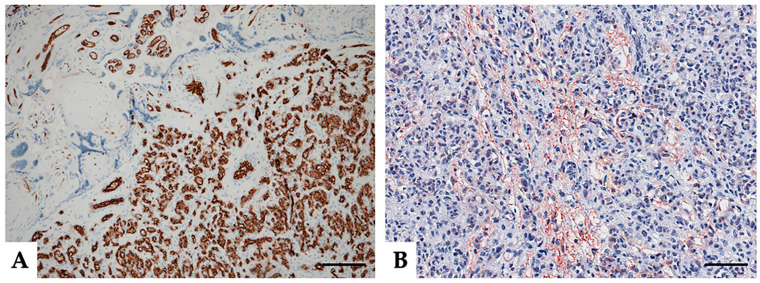

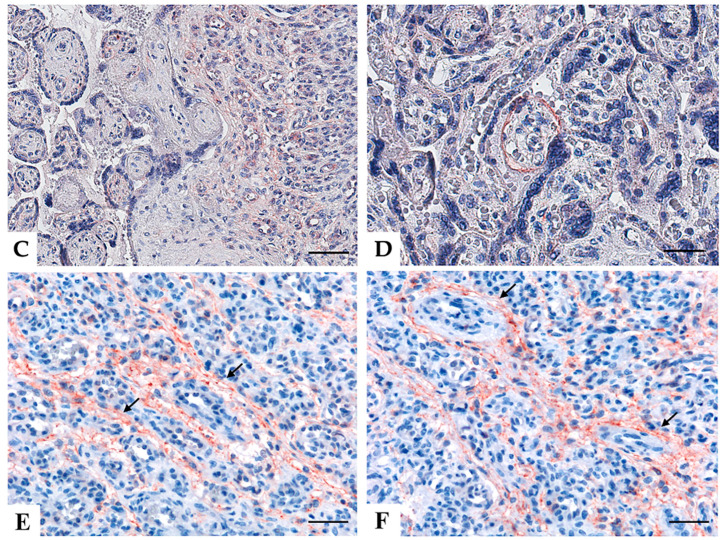
The pattern of immunohistochemical expression of CD31 and CD133 in placental chorioangioma. CD31 immunostaining showing vascular endothelium ((**A**) magnification 100×, scale bar 200 μm). Representative microphotographs relative to CD133 expression within tumoral contexture ((**B**) magnification 200×, scale bar 100 μm) and detailing the abrupt transition between the normal placenta and chorioangioma ((**C**) magnification 200×, scale bar 100 μm) and villi at higher magnification (black arrows) ((**D**–**F**) magnification 400×, scale bar 50 μm). Stains were revealed via DAB (3,3’-diaminobenzidine) or AEC (3-amino-9-ethylcarbazole, Dako, Denmark) substrate chromogen. Slides were counterstained with Harris Hematoxylin (Novocastra, Leica Biosystems).

Endothelial cells were diffusely immunoreactive for CD31 (also named platelet endothelial adhesion molecule 1, PECAM-1) (clone JC70—Cell Marque, Rocklin, CA, USA) (Figure 2A). Placental villi showed a regular vascular pattern in their axis, only occasionally showing the crowded capillaries that are most often observed in chorioangiosis. Immunostaining for CD133 (clone D2V8Q—Cell Signaling Technology, Inc., Danvers, MA, USA) was performed in multiple samples obtained from chorioangioma and placental parenchyma (Figure 2B,C). Cells of the chorioangioma showed evident diffuse membranous and cytoplasmatic expression of CD133, differently from the villi that exhibited a focal pattern of staining (Figure 2D). In particular, CD133 was expressed in syncytiotrophoblast and trophoblast elements (Figure 2D–F).

## 3. Discussion

Placental chorioangioma is the most common non-trophoblastic benign neoplasm of the placenta. Chorioangiomas occur in about 1% of all pregnancies [6,8,9,10] and they are considered the most common benign tumors of the placenta, even if they are sometimes classified as placental hamartoma [14]. Chorioangiomas are believed to arise since the 16th day of fertilization, although no one has documented it yet [15]. Chorioangiomas are associated with maternal age, hypertension, diabetes, female sex of newborn, premature labors, first delivery and multiple pregnancies [16,17,18].

Small chorioangiomas (<5 cm) are usually asymptomatic and are usually not diagnosed or incidentally found at histological examination [19], while larger tumors are more often correlated with maternal and fetal complications as preeclampsia, placental abruption, fetal congestive heart failure, haemolytic anaemia, congenital anomalies, fetal thrombocytopenia, cardiomegaly [20,21,22,23,24]. Larger chorioangiomas are mostly associated with fetal growth restriction, preterm delivery and stillbirth [23,24]. Other complications include polyhydramnios and fetal hydrops due to the high-output heart failure related to arteriovenous shunting [5,25] and disseminated intravascular coagulation due to platelet sequestration within the chorioangioma [26]; sometimes it may also spontaneously regress leading to the subsiding of symptoms [5].

Chorioangioma can sometimes be confused at ultrasound examinations with placental teratoma, degenerated myoma and blood clot [15], but histologically they can be easily recognized. In fact, a blood clot is easily identified in regular sections; the presence of all 3 germ layers is generally representative of mature teratoma [27,28] and finally degenerated leiomyomas formed by smooth muscle bundles separated by well-vascularized connective, with areas of degeneration including hyaline or mucoid change, calcification, cystic change or fatty metamorphosis; nevertheless, the tissue is positive to smooth muscle actin, desmin, H-caldesmon and vimentin [10,29]. The most important histological conditions taken into consideration for a differential diagnosis include: (A) chorioangiosis and chorioangiomatosis, which manifest as diffuse or more often as a focal proliferation of villous angioblastema, showing characteristic villi that are usually absent in chorioangioma [30]; (B) chorioangioma with trophoblast proliferation which is a rare proliferation of trophoblastic tissue in close association with chorioangiomas [5].

We present an unusual case of giant chorioangioma in a second gravida nulliparous woman at the 35th week of pregnancy. Clinically, most of the large or giant chorioangiomas are symptomatic and they can lead to pregnancy disorders, ranging from vaginal bleeding to poor fetal outcome and severe intrapartum complications in the most serious cases. The case in examination was asymptomatic, being one of the few clinical exceptions to what is reported in the literature [31,32], and the ultrasound test was negative. The placenta presented a normal weight, no diagnosis was done prenatally, and the baby did not show any of the impairments listed above. The chorioangioma was discovered only by histological examination of the placenta.

In terms of clinical implications, a review of 2010 confirmed the high rate of fetal complications in large or giant chorioangiomas [19]. Differently from the cases reported in the literature, where usually a fetal or neonatal unfavorable outcome was associated with this kind of disease, our case ended with a well-grown and healthy newborn, despite the lack of a pre-birth diagnosis. In fact, although large in size, the mass did not create any disorder during pregnancy and the newborn did not show intrapartum and postpartum complications. A possible explanation for the favorable outcome can be found in the paracentral and intraparenchymal position of the nodule, which enabled the gradual growth of the mass in a symbiotic way with the placenta, without interfering with the remaining placental vascularization and therefore without compromising the nourishment of the fetus. Probably the healthy part of the remaining placenta and its vascularization represented a sufficient *reservoir* for the blood supply favoring the symbiotic growth of the fetus and the lesion, without any manifestation of the latter. The fever that the patient had at the beginning was probably caused by a possible sub-clinical infection, then spontaneously regressed.

Histologically, the mass was defined as a cellular chorioangioma because it showed numerous small vessels within a relatively scant stroma with the presence of coarse calcifications. The neoplasm does not seem to depend on the maternal estro-progestin hormone state, as opposed to other gynecological vascular lesions that express estrogen. However, interestingly the tumor cells in the vessels showed widespread positivity to CD133, a typical marker of cancer stem cells [33]. CD133 has been studied in malignant tumor cells of different organs assuming a prognostic role, being involved in tumor growth; also, it contributes to shorter survival, tumor progression and tumor recurrence [34,35]. The expression of CD133 is controlled by many extracellular or intracellular factors, such as tumor microenvironment, epigenetic factors, signaling pathways, and mRNAs [36]. CD133 has been also found in congenital hemangiomas, congenital vascular malformations and placenta villi suggesting that the CD133^+^ precursor cells may be the source of endothelial cells underlying the congenital vascular malformation [37]. On the contrary, the expression of CD133 in placental chorioangiomas has never been described before. The widespread positivity in chorioangioma that we observed in our case suggests the presence of a rich component of stem cells among the cells composing the lesion and could explain its growth to large dimensions. The rich staminality could have promoted uncontrolled growth, possibly as a result of estrogen and progestin stimulation, as described in vascular neoplasms of the gynecological tract [38]. The presence of stem cells could be responsible for the growth of vascular structures undergoing a trophoblastic-mesenchymal transformation towards a vascular phenotype, with the involvement of multi-potent cells. In conclusion, stem cells could become a therapeutic target to counter the growth of vascular lesions in order to reduce the related risks of pregnancy disorders.

## 4. Conclusions

In this paper we would like to highlight the characteristics of a clinical unexpected manifestation of a giant chorioangioma and to report the observed widespread positivity of CD133, suggesting a pivotal involvement of stem cells in its formation. The identification of these cells, likely responsible for the growth of vascular structures presumably through a switch from a trophoblastic-mesenchymal phenotype to a vascular phenotype, may allow the identification of new possible future applications of CD133 as a histological marker of chorioangiomatous lesions as well as new therapeutic targets in order to decrease pregnancy complications. Nevertheless, the prenatal screening of placental vascularization may help to select the population of pregnant women who need a strict follow-up in order to identify earlier fetal impairment.

## Data Availability

The data presented in this study are available within the article.

## References

[1] Redline R.W. (2015). Classification of placental lesions. Am. J. Obstet. Gynecol..

[2] Redline R.W., Bagby C., Ravishankar S., Khong T.Y., Mooney E.E., Nikkels P.G.J., Morgan T.K., Gordijn S.J. (2019). Hypervascularity. Pathology of the Placenta: A Practical Guide.

[3] Carlucci S., Stabile G., Sorrentino F., Nappi L., Botta G., Menato G., Masturzo B. (2020). The singular case of multiple chorangioma syndrome in an IVF pregnancy. Analysis of the case and review of literature. Placenta.

[4] Carlucci S., Stabile G., Catagini S., Borghi C., Scutiero G., Morano D., Greco P. (2020). Fetal disseminated intravascular coagulopathy, hydrops and massive umbilical vein thrombosis consequence of a rare placental condition: Multifocal chorangiomatosis. J. Matern. Fetal Neonatal Med..

[5] Amer H.Z., Heller D.S. (2010). Chorangioma and related vascular lesions of the placenta—A review. Fetal Pediatr. Pathol..

[6] Yadav M., Maheshwari M., Sharma S., Godha Z., Garg P., Sharma G. (2017). Chorioangioma of Placenta: A Rare Case of Near-Miss Mortality. J. Obstet. Gynaecol. India.

[7] Benirschke K., Kaufmann P. (1995). Benign Tumors. Pathology of the Human Placenta.

[8] Sethi S., Hemal U., Solanki R., Bhagra A. (2004). Chorioangioma of placenta: A case report. Indian J. Radiol. Imaging.

[9] Lobo-Antunes I., Vinagre C., Rodrigues M., Ilgenfritz R., Bernardo A., Santos A. (2017). Spontaneous necrosis of a giant placental chorioangioma: Case report. Reprodução Clim..

[10] Miliaras D., Anagnostou E., Papoulidis I., Miliara X. (2011). Non-trophoblastic tumor of the placenta with combined histologic features of chorangioma and leiomyoma. Placenta.

[11] Marchetti A.A. (1939). A consideration of certain types of benign tumors of the placenta. Surg Gynecol. Obstet..

[12] Grundy H.O., Byers L., Walton S., Burlbaw J., Dannar C. (1986). Antepartum ultrasonographic evaluation and management of placental chorioangioma. A case report. J. Reprod. Med..

[13] Fox H. (1978). Non-trophoblastic tumours of the placenta. Pathol. Placenta.

[14] Köhnel P. (1933). Placental chorioangioma. Acta Obstet. Gynecol. Scand..

[15] Bracero L.A., Davidian M., Cassidy S. (1993). Chorioangioma: Diffuse angiomatous form. Chorioangioma.

[16] Hirata G.I., Masaki D.I., O’Toole M., Medearis A.L., Platt L.D. (1993). Color flow mapping and Doppler velocimetry in the diagnosis and management of a placental chorioangioma associated with nonimmune fetal hydrops. Obstet. Gynecol..

[17] Guschmann M., Henrich W., Entezami M., Dudenhausen J.W. (2003). Chorioangioma—New insights into a well-known problem. I. Results of a clinical and morphological study of 136 cases. J. Perinat. Med..

[18] Guschmann M., Henrich W., Dudenhausen J.W. (2003). Chorioangiomas—New insights into a well-known problem. II. An immuno-histochemical investigation of 136 cases. J. Perinat. Med..

[19] Zanardini C., Papageorghiou A., Bhide A., Thilaganathan B. (2010). Giant placental chorioangioma: Natural history and pregnancy outcome. Ultrasound Obstet. Gynecol..

[20] Tan S.A., Yeo S.H. (1992). Placental chorioangioma: A case report and review. Singapore Med. J..

[21] Kodandapani S., Shreshta A., Ramkumar V., Rao L. (2012). Chorioangioma of placenta: A rare placental cause for adverse fetal outcome. Case Rep. Obstet. Gynecol..

[22] Abdalla N., Bachanek M., Trojanowski S., Cendrowski K., Sawicki W. (2014). Placental tumor (chorioangioma) as a cause of polyhydramnios: A case report. Int. J. Womens Health.

[23] Mucitelli D.R., Charles E.Z., Kraus F.T. (1990). Chorioangiomas of intermediate size and intrauterine growth retardation. Pathol. Res. Pract..

[24] Fan M., Skupski D.W. (2014). Placental chorioangioma: Literature review. J. Perinat. Med..

[25] Ogino S., Redline R.W. (2000). Villous capillary lesions of the placenta: Distinctions between chorangioma, chorangiomatosis, and chorangiosis. Hum. Pathol..

[26] Jones C.E., Rivers R.P., Taghizadeh A. (1972). Disseminated intravascular coagulation and fetal hydrops in a newborn infant in association with a chorangioma of placenta. Pediatrics.

[27] Wang L., Du X., Li M. (1995). Placental teratoma. A case report and review of the literature. Pathol. Res. Pract..

[28] Reus W.A., Geppert M. (1988). Teratoma of the placenta. Geburtshilfe Frauenheilkd.

[29] Bello L.V. (2018). Placentary chorangio-leiomyoma. Medicina (B Aires).

[30] Lez C., Fures R., Hrgovic Z., Belina S., Fajdic J., Munstedt K. (2010). Chorangioma placentae. Rare Tumors.

[31] Kataria N., Singh A., Bedi P.K. (2016). Giant Placental Chorangioma: A Rare Case Report. J. Clin. Diagn. Res..

[32] Ting E.L.P., Yong S.L., Suhashini G., Kang M. (2019). A mystery mass on the placenta. Horm. Mol. Biol. Clin. Investig..

[33] Wu Y., Wu P.Y. (2009). CD133 as a marker for cancer stem cells: Progresses and concerns. Stem Cells Dev..

[34] Reggiani Bonetti L., Migaldi M., Caredda E., Boninsegna A., Ponz De Leon M., Di Gregorio C., Barresi V., Scannone D., Danese S., Cittadini A. (2012). Increased expression of CD133 is a strong predictor of poor outcome in stage I colorectal cancer patients. Scand. J. Gastroenterol..

[35] Coco C., Zannoni G.F., Caredda E., Sioletic S., Boninsegna A., Migaldi M., Rizzo G., Bonetti L.R., Genovese G., Stigliano E. (2012). Increased expression of CD133 and reduced dystroglycan expression are strong predictors of poor outcome in colon cancer patients. J. Exp. Clin. Cancer Res..

[36] Aghajani M., Mansoori B., Mohammadi A., Asadzadeh Z., Baradaran B. (2019). New emerging roles of CD133 in cancer stem cell: Signaling pathway and miRNA regulation. J. Cell Physiol..

[37] Li N., Wang Y.R., Zhong A.M., Wang W.J. (2007). Differential expression of CD133, Glut-1 in tissues and endothelial cells derived from infantile hemangioma and vascular malformation. Zhonghua Zheng Xing Wai Ke Za Zhi.

[38] Reggiani Bonetti L., Boselli F., Lupi M., Bettelli S., Schirosi L., Bigiani N., Sartori G., Rivasi F. (2009). Expression of estrogen receptor in hemangioma of the uterine cervix: Reports of three cases and review of the literature. Arch Gynecol. Obstet..

